# From Chemotherapy to Combined Targeted Therapeutics: *In Vitro* and *in Vivo* Models to Decipher Intra-tumor Heterogeneity

**DOI:** 10.3389/fphar.2018.00077

**Published:** 2018-02-14

**Authors:** Guido Gambara, Manuela Gaebler, Ulrich Keilholz, Christian R. A. Regenbrecht, Alessandra Silvestri

**Affiliations:** ^1^Charité Comprehensive Cancer Center, Charité – Universitätsmedizin, Berlin, Germany; ^2^German Cancer Consortium (DKTK), German Cancer Research Center (DKFZ), Heidelberg, Germany; ^3^Department of Interdisciplinary Oncology, HELIOS Klinikum Berlin-Buch GmbH, Berlin, Germany; ^4^cpo - Cellular Phenomics & Oncology Berlin-Buch GmbH, Berlin, Germany

**Keywords:** cancer models, PD3D cell culture, organoids, tumor heterogeneity, tumor microenvironment, tumor evolution, cancer stem cells, PDX models

## Abstract

Recent advances in next-generation sequencing and other omics technologies capable to map cell fate provide increasing evidence on the crucial role of intra-tumor heterogeneity (ITH) for cancer progression. The different facets of ITH, from genomic to microenvironmental heterogeneity and the hierarchical cellular architecture originating from the cancer stem cell compartment, contribute to the range of tumor phenotypes. Decoding these complex data resulting from the analysis of tumor tissue complexity poses a challenge for developing novel therapeutic strategies that can counteract tumor evolution and cellular plasticity. To achieve this aim, the development of *in vitro* and *in vivo* cancer models that resemble the complexity of ITH is crucial in understanding the interplay of cells and their (micro)environment and, consequently, in testing the efficacy of new targeted treatments and novel strategies of tailoring combinations of treatments to the individual composition of the tumor. This challenging approach may be an important cornerstone in overcoming the development of pharmaco-resistances during multiple lines of treatment. In this paper, we report the latest advances in patient-derived 3D (PD3D) cell cultures and patient-derived tumor xenografts (PDX) as *in vitro* and *in vivo* models that can retain the genetic and phenotypic heterogeneity of the tumor tissue.

## Introduction

Tumor subtyping across different patients is based on multiple cancer features, such as genomic landscape, biomarker expression, and morphology, and it is routinely used in clinical settings. These characteristics contribute to the definition of cancer phenotypes and ultimately to the categorization of tumors in different stages and grades. At present, the design of targeted therapies is mainly based on an understanding of the above described inter-tumor (inter-patient) heterogeneity, although the presence of heterogeneity within an individual tumor is broadly recognized. Increasing evidence suggests that intra-tumor heterogeneity (ITH) is clinically relevant, as proven by the mutable “status” of molecular biomarkers in time and by the acquisition of distinct phenotypes diverse from the primary tumor in metastatic lesions (Bedard et al., 2013). Russo et al. (2016) have recently shown that a biopsy from a single lesion can be inadequate for the selection of a targeted therapy directed at the treatment of multiple metastases within the same patient. The multifaceted aspects of intra-tumor heterogeneity include genetic and epigenetic heterogeneity as well as phenotypic heterogeneity derived from the cellular hierarchy originating from the cancer stem cell compartment and microenvironment heterogeneity (De Sousa et al., 2013). These components are strictly connected and interdependent, participating in the temporal and spatial complexity and mutability of tumor phenotypes. Independently from the underlying mechanisms that lead to ITH, the fact that multiple cell populations exist in the same tumor has strong clinical implications. Patient-specific responses to therapy and the development of resistance to chemo- and targeted therapies are the direct results of ITH (Diaz et al., 2012).

Two main factors are responsible for ITH: (1) intrinsic clonal dynamics in a cell’s genetic or epigenetic background and (2) tumor-extrinsic factors in the non-cell autonomous compartment. Cleary et al. (2014) have demonstrated that clonal populations within the tumor exist dynamically in time and space, competing with and eventually supporting one another, and the entirety of this population defines the properties of the tumor as a whole. Through the entire course of tumor development and progression, malignant transformed cells constantly undergo mutational events that in an evolutionary fashion underlie a trial and error principle, which may or may not result in a selective advantage relative to neighboring cells. However, the selection process occurs at least in part as a response to stressors, which may be stable or transient (Gupta et al., 2011). Therefore, a gain of fitness in one clone relative to another does not necessarily lead to the loss of the latter in favor of the former. Extrinsically, the major factor contributing to ITH is cellular interactions with the extracellular matrix (ECM), which can alter gene expression, thus driving differentiation and altering cell properties. It is possible that altered cell matrix contacts are essential for the stability of clonal composition within a tumor (Marusyk et al., 2012).

Not only the ECM but also the broader tumor microenvironment with its cellular stromal components influences ITH. Infiltrating cancer-associated fibroblasts (CAF) are described as resistant to cytotoxic and targeted therapies (Straussman et al., 2012), and only lately studies have shown that these fibroblasts demonstrate a certain degree of plasticity themselves (Augsten, 2014). The composition of the tumor bulk in its variety of tumor cell clones and the infiltrating CAF cause a broad range of responses toward systemic and targeted treatment and can be in part responsible for the constant failure of reliable predictions about treatment success. Therefore, in this review, we first highlight the impact of ITH in clinical settings, analyzing the consequences of ITH on patient management and alternative therapeutic approaches potentially able to overcome tumor evolution. Then, since tumor models able to recapitulate different aspects of intra-tumor heterogeneity are crucial in allowing researchers to decipher the dynamics of cancer phenotypes and reshape therapeutic strategies, we shortly introduce the different sources of ITH and subsequently focus on the capability of patient-derived 3D (PD3D) cell cultures and patient-derived tumor xenografts (PDX) to illustrate different facets of ITH.

## Clinical Relevance and Exploitation of Intra-Tumor Heterogeneity

The emergence of sophisticated “omic” technologies has boosted our understanding of the molecular events underlying cancer development and progression. Despite these findings, the development of new compounds often fails in the transition from preclinical to clinical stages, which to a certain extent is owed to preclinical models insufficiently recapitulating the complexity of solid cancers. Data from the EMA and the FDA suggest that new targeted drugs show an effect in 10–20% of a non-stratified cohort, while proper stratification almost doubles therapy efficiency. Huang et al. (2014) have published similar numbers. As increasing tumor sequencing data becomes available from large cohorts within the framework of multinational sequencing projects such as the ICGC and the Cancer Genome Atlas, researchers have identified more molecular subtypes of tumors (Curtis et al., 2012; Guinney et al., 2015; Gaebler et al., 2017). The use of cetuximab-based therapy in RAS wildtype colorectal cancers (Khambata-Ford et al., 2007) and the use of ALK kinase inhibitors in EML4-ALK-positive NSCLC (Shaw et al., 2013) are two successful examples. Nevertheless, even with appropriate companion diagnostics, such targeted therapies often add only 6–12 months of progression-free survival until the patient presents with disease progression (Huang et al., 2014). For this reason, researchers and pharmaceutical groups have turned to patient-derived organoids (Silvestri et al., 2017) or patient-derived xenograft models (Hidalgo et al., 2014; Whittle et al., 2015).

Tumors are highly adaptive to their (micro)environments. Given this, understanding the mechanisms underlying tumor development and clonal evolution will help researchers predict their evolutionary trajectories, with direct implications for the choice of therapeutic interventions. Since liquid biopsies entered clinical routines, serial sampling of tumor genomes with minimal intervention has helped researchers understand the evolution of drug-resistance mechanisms over time (Haber and Velculescu, 2014) as well as the evolution of metastatic diseases (Carreira et al., 2014). Also, analyses of micro vesicles and exosomes from tumors found in patient serum have proven useful in allowing researchers to predict the sites of future metastases for certain tumor entities (Zarour et al., 2017). Any clonal event that drives tumorigenesis may pose an attractive target model for drug development. As such, the benchmark for successful clinical trials in the approval process is progression-free survival in direct comparison to the standard of care. Given the failure of targeted therapies in advanced disease without prior chemotherapy, it is likely that many targeted therapies have achieved this benefit for patients by targeting early clonal events (e.g., driver mutations) present in the majority of cells. Unfortunately, in the clinical context, resistance to such therapies is frequently observed. Residual tumor cells remain viable after chemotherapy as a result either of the selection of resistant clones present at low frequencies in the treatment-naive tumor (Turke et al., 2010; Su et al., 2012; Bhang et al., 2015) or of acquired mutations during therapy (Hata et al., 2016).

Bozic and Nowak (2014) have estimated that upon diagnosis, tumor lesions harbor at least ten or more sub-clones that resist monotherapy. Consequently, single targeted agents are unlikely to effectively kill all tumor cells. Targeted agents need to be combined to collectively act through inhibition of distinct pathways (Bozic et al., 2013). However, in practice, too little data is available about the synergistic and toxic effects of combinatory treatments and the biological consequences of the interplay of the mutational landscape in the tumor, limiting the use of such treatments in clinical settings. Therefore, the current focus of preclinical, translational, and pharmaceutical research is to exploit the immune system. Vaccine or adaptive T-cell therapy approaches targeting multiple clonal neo-antigens minimize normal tissue toxicity and maximize tumor cell kill while limiting the possibility for acquired drug resistance. It remains to be seen to what extent tumors are able to circumvent such strategies by means of DNA repair defects, resulting genomic instabilities, and thereby the loss of neo-antigens.

Genomic instability leads to cell-to-cell variation, fueling selection, plasticity, and evolution. The clinical relevance of genomic instability is reflected by the outcomes across multiple cancer types and evidence linking chromosomal chaos with metastasis (Turajlic and Swanton, 2016). Targeting mechanisms underlying genomic instability may limit disease progression, particularly in the early stages. One example of the successful targeting of unstable cancers is the success of PARP inhibitors in the treatment of *BRCA* mutant cancers. These inhibitors increase genomic instability to lethal levels, resulting in synthetic lethality (Lord and Ashworth, 2016). However, even with this sophisticated therapeutic regimen, resistance can occur either directly through additional mutations to *BRCA* or indirectly through, for example, inactivation of 53BP1 (Lord and Ashworth, 2016). Interestingly, Carey et al. (2017) have recently demonstrated that the efficacy of PARP inhibitors in MYC-driven triple-negative breast cancer cells can be further increased by concomitant downregulation of MYC expression using the cyclin-dependent kinase inhibitor dinaciclib. These data highlight the importance of deeply characterizing the entire tumor mass not only at tumor diagnosis but also during therapy to detect any new occurring alterations that could pose potential targets for adaptive therapies.

Evolutionary studies have revealed distinct mutagenic processes that occur through the disease course, best studied in the context of colorectal cancer (CRC) by Fearon and Vogelstein (1990). However, evidence is also emerging in lung adenocarcinoma, bladder cancer, estrogen receptor negative breast cancer, head and neck squamous carcinoma, and esophageal squamous carcinoma (Faltas et al., 2016; Law et al., 2016). Progression-free survival (PFS) times as commonly reported in clinical trials rarely translate to equivalent clinically relevant overall survival benefits (Fojo et al., 2014). Preclinical data from strictly stratified, well-characterized models does not hold up with the variations and complexities of a clinical trial. Progression-free survival frequently does not match overall survival, and this evidence may reflect ITH effects. If, for example, a dominant drug-sensitive clone is effectively targeted in the investigational arm of a trial, this may allow resistant sub-clones to undergo accelerated growth in a resource-rich environment. Ultimately, this results in a more aggressive and rapid disease progression compared to that in the control arm of the trial.

To estimate the efficacy of new drugs, researchers must design new trial concepts to allow for a more representative comparison to current therapies. For this reason, Gatenby et al. (2009) have designed study protocols in preclinical models that take into account the influence of resistant sub-clones by maintaining a stable population of sensitive clones (Enriquez-Navas et al., 2016). In contrast to standard clinical practice, where the goal of therapy is to maximally reduce tumor burden, the focus of adaptive therapy is to maximize time to progression by stabilizing tumor size (Gatenby et al., 2009; Enriquez-Navas et al., 2016). Adaptive therapy is based on a two-phased strategy: (1) an induction phase to avoid exponential tumor growth and (2) a maintenance phase using progressively lower doses, potentially including omitted schedules. In certain clinical settings, this strategy could achieve better progression-free survival times compared to standard fixed dosing. For example, in melanoma PDXs, Stuart and colleagues demonstrated how vemurafenib-resistant melanomas can acquire drug dependency such that an intermittent rather than continuous dosing of the drug can delay the onset of insuperable drug resistance (Das Thakur et al., 2013).

Traditional approaches to cancer management have primarily focused on overcoming drug resistance through multiple lines of treatment. In a more proactive approach, the tumor’s “next move” would become predictable through an understanding of evolutionary mechanisms and through the exploitation of evolutionary constraints or synthetic lethality. In renal cell carcinomas, Voss et al. (2014) examined five cases in which patients had experienced a prolonged benefit from mTOR pathway inhibition as a result of the use of everolimus or temsirolimus. Multi-region tumor sampling, as first suggested by Gerlinger et al. (2012), revealed parallel evolution of distinct somatic mutations, leading to activation of the mTOR pathway in independent areas of the tumor in three of the five cases investigated (Voss et al., 2014). Although rather anecdotal, these data suggest that targeting constraints to tumor evolution might be practical if appropriate biomarker assays and preclinical models are available. Collateral sensitivity, the phenomenon through which acquired resistance to one drug comes at the expense of sensitivity to another (Hill, 1986; Jensen et al., 1997), is another example of how ITH can be exploited for successful treatment by applying treatments aimed at reducing karyotypic heterogeneity until a defined predictable state through this initial drug exposure is reached. At this level, cells become susceptible to a secondary drug. For example, Bardelli and colleagues have demonstrated that KRAS mutant sub-clones of colorectal cancers are more sensitive to withdrawal of an EGFR monoclonal antibody-based regimen such as cetuximab than are their wildtype counterparts. This suggests an evolutional disadvantage after the acquisition of KRAS mutations in later tumor evolution, providing an explanation for further tumor response in later treatment lines (Siravegna et al., 2015).

Zhao et al. (2016) have shown in murine models of Philadelphia chromosome-positive ALL that collateral sensitivity is induced by treatment with dasatinib, leading to acquired resistance in the form of selection toward the BCR-ABL1 V299L mutation during the evolution of Ph^+^ ALL cells. This in turn generates cells sensitive to non-classical BCR-ABL inhibitors such as cabozantinib and vandetanib.

Considering that the development of distant metastasis is the main cause of death among cancer patients and that ITH strongly influences tumor response to treatment, several groups have focused their attention on profiling secondary tumor masses to not only better understand tumor evolution but also define better treatments for metastatic patients. Tumor metastases can in fact be characterized by different molecular profiles compared to that of the primary mass, and this heterogeneity might underlie the limited therapeutic success in metastatic patients when treatment is based only on the prognostic signature of the primary tumor.

In pancreatic cancer, Campbell et al. (2010) have shown that amplification in cancer genes occurs predominantly in the early steps of cancer development and that genomic instability persists during cancer dissemination, with a consequent parallel and even convergent evolution among different metastases. Moreover, this study showed that metastasis-initiating cells are characterized by genetic heterogeneity and that it is possible to define organ-specific branches in the phylogenetic trees across metastases. This specificity can be caused by the presence of particular genotypes that drive metastatic cells to specific organs and/or by sub-clones whose rearrangements make them more suitable for survival in a specific secondary organ. Heterogeneity between primary lesions and distant metastasis has been demonstrated not only in pancreatic cancer but also in other tumor types, such as breast cancer (Ng et al., 2017) and salivary adenoids cystic carcinoma (Liu et al., 2017).

Heterogeneity between the primary tumor and distant metastasis has been shown not only at the genomic level but also considering protein activation (Silvestri et al., 2013). In particular, a higher activation of the EGFR-PDGFR-cKIT network, in addition to the PI3K/AKT pathway, has been demonstrated in liver metastasis compared to primary colorectal cancer tumors. Interestingly, recent work has shown that when different metastatic masses from the same patient are analyzed, heterogeneity among them is limited. In particular, Makohon-Moore et al. (2017) have highlighted the presence of identical mutations in well-known driver genes while the ITH is related only to passenger gene mutations without any functional consequences. The limited heterogeneity characterizing secondary masses has been shown also by the consistency between protein pathway activation profiling of different regions of colorectal cancer liver metastases (Parasido et al., 2017). Taken together, these data have important clinical implications, highlighting the importance of secondary mass profiling for therapy definition when the metastasis is the target of the therapy. Moreover, if validated in clinical studies, recent data highlighting limited heterogeneity in metastatic masses have encouraging implications for the future success of therapies for metastasis.

## Cell Culture Models of Regional Genomic Architecture and Intra-Tumor Heterogeneity

Tumor evolution driven by genetic variations is surely the most largely investigated source of inter- and intra-tumor heterogeneity. The genetic component of intra-tumor heterogeneity derives from selective pressure that allows the growth of distinct clones. These clones interact with one another within the same tumor. It is this interaction that promotes or inhibits cell growth and often confers therapy resistance in response to therapeutic interventions (Greaves and Maley, 2012). Nowell (1976) introduced a model defining the branching architecture of clonal evolution in cancer initiation and propagation, describing this as a process through which the mutation of a “progenitor normal cell” induces an advantage in cell growth that determine the prevalence of the cancer clone among the normal cell population in the tissue. Genomic instability within this population can subsequently generate other populations that, following Darwinian selection, occasionally generate new leading sub-clones with a phenotypic advantage (Nowell, 1976). Mutations leading clonal evolution are essentially driven by system defects that maintain genome integrity (DNA repair mechanisms), exposure to genotoxic agents, or treatment with cytotoxic therapies. Thus, genomic instability results in the accumulation of a high number of different genomic aberrations, from single-point mutations to small insertions and deletions, chromosomal rearrangement, or doubling of the entire genome (Burrell et al., 2013). Random accumulation of these aberrations results in a multitude of phenotypes controlled by the constant selective pressure of the “healthy” tissue habitat. In this context, the cancer microenvironment retains a tumor-suppressive role, especially in cancer onset, but the reciprocal interaction of the microenvironment and malignant cells contributes to this dynamic modeling, which generates niches favorable to tumor growth (De Palma et al., 2005; Lathia et al., 2011). In this view, genetic differences between sub-clones of cancer cells, found mostly between the primary and metastatic site, can be in part explained by the different selection induced by the distinctive microenvironments at both sites.

Increasing evidence shows that different cancer cell populations resembling individual genetic clones within the same tumor can evolve in parallel (allopatric speciation), especially if physically separated within the tumor, or interact through cooperative (symbiotic evolution) or competitive (antagonist evolution) behavior (McGranahan and Swanton, 2015). Hobor et al. (2014) have observed an example of clonal cooperation in colorectal cancer, where the growth of KRAS wt sub-clones, usually sensitive to cetuximab, are supported by KRAS mutant sub-clones. Marusyk et al. (2014) have likewise observed that tumor growth can be supported by sub-clones that do not necessarily have enhanced fitness but can impair microenvironment integrity and consequentially improve the non-cell autonomous growth of polyclonal tumors. In addition, the authors have demonstrated that the interaction of different sub-clones, overexpressing secreting factors, can develop new phenotypic traits, such as metastatic and hemorrhage features.

As regards multiregional sampling and analyses, the exome sequencing of samples obtained through a spatially separated biopsy of primary renal carcinomas and metastasis shows a high percentage of somatic mutation not detectable in different areas of the same tumor. Intra-tumor heterogeneity shows on one hand the presence of different phenotypes within the tumor (mTOR mutation) or, on the other, heterogeneous mutation sites in the same genes (SETD2, KDM5 and PTEN), indicating a phenotype convergence in the evolution of the tumor (Gerlinger et al., 2012). Moreover, multiregional sequencing shows that in clear cell renal carcinoma, intra-tumor driver genomic aberrations are delimited to spatially separated sub-clones, and their numbers increase with the number of samples analyzed without saturation, suggesting that the analysis of a single biopsy can largely underestimate the prevalence of driver mutations in a patient (Gerlinger et al., 2014).

Recently, it has been shown that next-generation sequencing combined with the establishment of patient-derived primary cell culture can improve the analysis of intra-tumor heterogeneity and help to overcome its consequences on the development of drug-resistant sub-clones. In this framework, Gao et al. (2017) studied intra-tumor heterogeneity in hepatocellular carcinoma (HCC), sequencing primary cancer cell cultures derived from different regions of tumors from 10 patients (for a total of 55 regions). Evidence of heterogeneous mutations (39.7%) and branched evolution were found in each tumor, and in the presence of targetable genomic aberrations, the sensitivity of the cell culture to the specific drugs was confirmed. Moreover, high throughput screening was performed to identify drugs able to affect the viability of resistant sub-clones. Interestingly, the authors demonstrated that cell cultures retained a mutational profile highly similar to the original tissue also at high passages, supporting the use of a long-term primary cancer cell culture as an *in vitro* model to assay the effect of intra-tumor heterogeneity on drug sensitivity. Gerber et al. (2017) have similarly investigated inter- and intra-tumor heterogeneity in primary cell cultures derived from three melanoma metastases from three different patients (BRAF wt/NRAS wt, BRAF mut/NRAS wt, and BRAF wt/NRAS mut). The authors analyzed inter- and intra-tumor heterogeneity by means of single cell RNA-seq, identifying CDK4, highly expressed in most of the sub-clones derived from BRAF wt/NRAS wt tumors, as a potential therapeutic target. Moreover, a small population of cells expressing the ABC transporter ABCB5, the stem cell markers CD271 and CD133, and aldehyde dehydrogenases (ALDH) was identified within the same tumor.

Few studies have investigated the ability of patient-derived 3D (PD3D) cell cultures to retain genetic intra-tumor heterogeneity. Weeber et al. (2015) have shown that 3D cell cultures (organoids) can be successfully generated from biopsies of colorectal cancer metastases and that these organoids retain 90% of somatic aberrations when compared with the original cancer tissues. The presence of discrepancies in 15 mutations have been attributed to intra-tumor heterogeneity. Giessler et al. (2017) have also investigated genetic sub-clone composition in colorectal cancer by means of whole genome sequencing of three patient tumors and the corresponding (short- and long-term) 3D cell cultures (spheroids) and xenografts. Analyzing small nucleotide variants and copy number alterations, the authors identified at least two genomic sub-clones coexisting in each primary tumor and at least three sub-clones in patient-derived spheroids and xenografts. Interestingly, these sub-clones were dynamic in both spheroids and xenografts, suggesting that different dominant sub-clones drive cell growth in these two models.

Van de Wetering et al. (2015) have generated a library of 22 patient-derived colorectal cancer organoids and compared the genomic landscape of these and the corresponding tissue biopsies, demonstrating that the polyclonal architecture of tumor biopsies is essentially maintained *in vitro*. Interestingly, a comparison of biopsies derived from two patients with two tumors each showed in one case the same alteration in TP53 R273C and BRAF V600E, indicating the derivation from a common progenitor and the subsequent divergence of sub-clones, while the biopsies obtained from the other patient showed different driving mutations in APC and P53. In both cases, the corresponding organoid cultures recapitulated the genomic landscape observed in the biopsies. Recently, Bruna et al. (2016) have also shown that patient-derived xenografts from breast cancer tissue and xenograft-derived cell cultures are able to preserve the intra-tumor genomic architecture of the original tumor tissue. In this study, the authors performed a drug screening using an *ex vivo* model consisting of both single cells and small tissue-derived cell aggregates (<40 μm). The effect of several drugs identified *in vitro* were also validated *in vivo*, demonstrating the reliability of this cell culture platform for pharmacogenomic studies.

The crucial role of a sub-population of cancer cells with stem-like properties has been largely investigated in the onset, growth, and propagation of glioblastoma (Galli et al., 2004; Lathia et al., 2015). These cells can be isolated from patient tissue as primary 3D neuro-spheres, propagated *in vitro*, and engrafted in immunodeficient mice. Using cytogenetic analysis and whole exome sequencing, Piccirillo et al. (2009) have shown that populations of glioblastoma cells with different genomic aberrations coexist within different regions of the same tumor, supporting the culture of glioblastoma neuro-spheres as a potent tool for the study of genetic intra-tumor heterogeneity and the evaluation of drug responses *in vitro* and *in vivo*.

**FIGURE 1:**
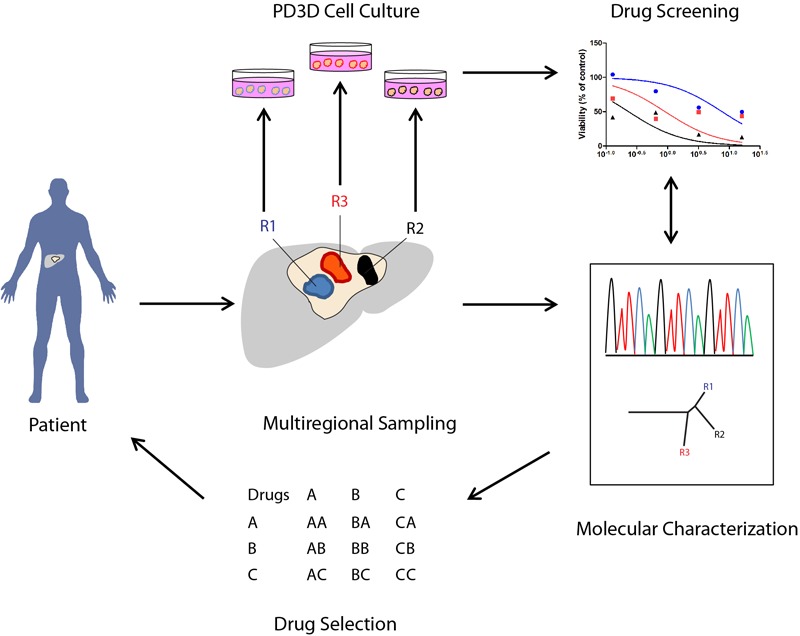
Potential applications of PD3D cell cultures derived from multiregional cancer biopsies in therapy selection (“Reverse Clinical Engineering”). The illustration shows how 3D cell cultures derived from multiregional sampling or biopsies can potentially improve the design of targeted therapies in cancer patients.

The model proposed in **Figure 1** recapitulates a potential workflow (“Reverse Clinical Engineering”) applicable for the development of a precision medicine platform, integrating multiregional sampling, molecular characterization, and PD3D cell culture drug testing to improve the design of targeted therapy and overcoming at least in part the consequences of intra-tumor heterogeneity in clinical scenarios. The feasibility of this approach has been previously proposed by Pauli et al. (2017), who have tested for drug resistance in PD3D and PDX models, integrating data derived from sequencing and histology of tumor tissues. In our opinion, the use of multisampling in space (biopsies from different tumor regions) or in time (serial biopsies in different time frames of tumor evolution) could improve the design of combined targeted therapies, overcoming ITH-derived drug resistances. Moreover, considering the need for less invasive diagnostic procedures, the use of liquid biopsies aimed at allowing the collection of circulating tumor DNA (ctDNA) (Russo et al., 2016) or circulating tumor cells (CTCs) could be a valid alternative, allowing researchers to follow tumor evolution in time and space, assuming the ctDNA and CTCs could represent multiple lesions.

## Cancer Stem Cell Compartments and Hierarchical Cellular Heterogeneity in PD3D Cancer Cell Culture Models

As previously described in the introduction paragraph, genetic, epigenetic, and phenotypic features contribute dynamically to the establishment of intra-tumor heterogeneity. For a long time, the clonal evolution model and the cancer stem cell model have competed in their explanations of the origins of tumor heterogeneity, but Kreso and Dick (2014) have recently shown that these two theories are highly complementary more than mutually exclusive. Within numerous malignant tissues, it has been shown that a small population of cells is responsible for the initiation, survival, and growth of the tumor. This population of cells, termed either cancer stem cells (CSCs) or tumor-initiating cells (TICs), has stem cell–like features and is responsible for the hierarchical organization of cells within the tumor, as is the normal stem cell counterpart in healthy tissue (Kreso and Dick, 2014). CSCs have a self-renewal capability (asymmetric division) and the ability to generate a progeny of cells that can undergo differentiation, thus generating the hierarchy observed in some cancer tissues (Batlle and Clevers, 2017). CSCs were initially identified in acute myeloid leukemia (AML) as a CD34^+^ CD38^-^ subpopulation capable of initiating leukemia after engraftment in immunodeficient mice (Lapidot et al., 1994). Subsequently, numerous studies in solid tumors have identified markers to define subpopulations of cancer cells able to initiate cancer and maintain the hierarchical organization in xenograft assays (Medema, 2013). CSC markers include CD44^+^/CD24^-/low^ in breast cancer (Al-Hajj et al., 2003), CD133^+^ in brain tumors (Singh et al., 2004) and CRC (Ricci-Vitiani et al., 2007), ALDH high in thyroid cancer (Todaro et al., 2010), CD44^+^/α2β1^high^/CD133^+^ in prostate cancer, and numerous markers in other solid tumors (Medema, 2013).

Cellular hierarchy can vary among different tumor entities: It can be organized in highly defined differentiation steps (steep hierarchy) or in a similar ratio between stem cells and differentiated non-tumorigenic cells (shallow hierarchy) (Meacham and Morrison, 2013). However, Roesch et al. (2010) have demonstrated that melanoma growth does not follow the typical hierarchical model; rather, the population of cells responsible for tumor heterogeneity is highly dynamic in time. In CRC, Francescangeli et al. (2015) have shown that Cripto-1 can dynamically regulate the cancer stem cell compartment *in vivo* and *in vitro* through Src/Akt signaling, suggesting that cancer cells can change their status from non-tumorigenic to tumorigenic and vice versa, occupying different places of the stemness hierarchy tree in different time frames. The ability of CSCs and more differentiated cells to dynamically change their phenotype is a prerogative of CSC plasticity. This mechanism is also involved in cancer onset; thus, Schwitalla et al. (2013) have demonstrated that differentiated intestinal epithelial cells within an inflammatory context undergo a dedifferentiation process led by Wnt that drives the acquisition of self-renewal stem-like features. Recently, Shimokawa et al. (2017) have studied CSC hierarchy and plasticity in human CRC-derived PD3D models by tracking *in vitro* and *in vivo* the expression of fluorescent markers located in the LGR5 (stem cells) or KRT20 (differentiated cells) locus. The authors showed that although the selective elimination of LGR5^+^ initially reduced tumor growth, the LGR5^+^ population subsequently reappeared, as demonstrated by the regrowth of the tumor. The authors suggest that differentiated CRC cells expressing KRT20 are able to generate LGR5^+^ cells only if the CSC niche is not occupied by the original LGR5^+^ cells.

Increasing evidence suggests that the CSC model principally contributes to intra-tumor heterogeneity by introducing functional diversity due to the coexistence of cells at different levels of the hierarchical tree within the bulk of tumor cells. Beyond the genetic alteration contributing to the clonal evolution of the tumor, described in the previous paragraph, the cancer epigenome has been shown to contribute to the establishment of different cellular phenotypes, representing the CSC-based hierarchical organization of the tumor (Easwaran et al., 2014). Both genetic and epigenetic alterations are strictly interconnected, since in different tumor entities it has been shown that mutation in genes coding for proteins involved in chromatin organization and DNA methylation are frequently represented (Shen and Laird, 2013). Moreover, the promoters of a high number of genes differentially regulated in cancer are known to be linked to chromatin, highly regulated in embryonic or adult stem cells. This chromatin, also called bivalent, is characterized by the presence of both repressive and active histone marks (respectively, H3K27me3 and H3K4me3) within not-methylated GpC island promoters and can switch between active or passive transcriptional activities during differentiation. The promoters of genes with bivalent chromatin, normally involved in embryonic development, are frequently hyper-methylated in cancer, altering the self-renewal feature of tumor cells, interfering with their differentiation, and contributing to the establishment of the CSC phenotype (Easwaran et al., 2012).

Torres et al. (2016) have demonstrated that in different tumors, the expression of the histone H1.0 is highly heterogeneous, and its epigenetically regulated silencing correlates with the self-renewal feature and tumorigenic ability, suggesting the rescue of H1.0 expression as a potential CSC targeted therapy. Recently, multiregional sampling of human glioblastoma followed by a transcriptomic profile by Jin et al. (2017) has shown that the perivascular and hypoxic tumor regions have two distinct gene expression programs associated with heterogeneity in the composition of glioblastoma stem cells (GSCs) derived from these two niches. Moreover, the authors have identified two chromatin modeling proteins, part of the polycomb-repressing complexes EZH2 and BMI1, as crucial players in the tumorigenic potential of the two different pools of GSCs. Finally, in this study, a therapy based on the combined inhibition of EZH2 and BMI1 was able to overcome the drug resistance caused by GSC heterogeneity.

Increasing evidence of the crucial role of CSC in tumor progression highlights the urgent need for sophisticated *in vitro* cancer models recapitulating the hierarchical structure observed in patient tissue to identify new efficient therapeutic approaches. In fact, Vermeulen et al. (2012) have demonstrated that the presence of CSC subpopulations is in part responsible for therapy resistance and cancer recurrence. In numerous tumor entities, treatment with cytotoxic drugs has been shown to enrich the CSC subpopulation, supporting the need to develop *in vitro* models capable of retaining cancer stem cells to test drug efficacy. It has been largely demonstrated that patient-derived 3D (PD3D) cell cultures (organoids or spheroids) are hierarchically organized cellular models suitable for the investigation of the impact of new therapies on cancer growth. For example, it has been shown that PD3D cell cultures obtained from colorectal cancer comprise self-renewing forming cells, low proliferating cells, and post-mitotic cells and that the percentage of these classes of cells is relatively stable in serial re-plating experiments, demonstrating that PD3D cell cultures maintain a linear functional cell hierarchy and that only a subclass of tumor-initiating cells (TICs) drives tumor onset, growth, and metastatic formation (Dieter et al., 2011). More recently, Giessler et al. (2017) have investigated the origin of this functional heterogeneity by analyzing primary CRC tumors, tumor-derived xenografts, and PD3D cell cultures by means of whole genome sequencing and functional assays. They showed that different genomic sub-clones were found in the original tumor and in both xenograft and PD3D cultures, demonstrating that TcICs (tumor clone-initiating cells) were genetically heterogeneous, contributing differently to tumor growth.

Recently, Fujii et al. (2016) have generated a library of organoids derived from 52 tumors with different subtypes and grades of colorectal cancer. Isolated organoids recapitulated the histotypes of the patients’ tumors, and a reduction in number of niche factors was observed in the transition from adenoma to carcinoma. Moreover, heterogeneity in gene expression within tumor organoids deriving from a single patient and between organoids deriving from different CRC patients has been analyzed by means of single organoid RNA-sequencing, showing that heterogeneity is present in both comparisons (van de Wetering et al., 2015). Finally, CRISPR/Cas9-based genome editing allows the tracing of human CRC PD3D models expressing EGFP under the control of an LGR5 promoter to follow the fate of CSCs *in vitro* and *in vivo*. These experiments have confirmed the hierarchical organization of CRC, showing the ability of CSCs to maintain their self-renewal properties as well as their capability to differentiate (Cortina et al., 2017).

As previously described, Singh et al. (2003, 2004) have been able to generate PD3D cell cultures (tumor spheres) from different pathological subtypes of brain tumors (medulloblastoma, astrocytoma, ganglioglioma, and ependymoma). PD3D cell cultures derived from these tumor entities retained self-renewal, proliferation, and differentiation capabilities, confirming their stem cell identity. Interestingly, isolated PD3D cell cultures were positive for stem cell markers such as CD133^+^ and nestin, but only when plated in differentiation media were they able to express neural differentiation markers. Moreover, the authors demonstrated that only the CD133^+^ subpopulation maintains its self-renewal capacity *in vitro*, and it is able to generate tumors in immuno-deficient mice that recapitulate the phenotypes of patient tumors. Recently, Hubert et al. (2016) have developed a novel 3D organoid culture system to recapitulate glioblastoma cancer stem cell heterogeneity. The authors showed that patient-derived cancer cells can be grown for months without passaging, generating organoids of variable size (in the range of millimeters) that contain a cancer stem cell compartment comparable with the culture of canonical glioblastoma-derived tumor spheres. Interestingly, organoids derived from multiple regions of the same astrocytoma can engraft with different efficiency in immunodeficient mice, reflecting the functionally different subpopulations present in different regions of the same tumor.

In breast cancer, Pece et al. (2010) have demonstrated that the signature of human normal mammary stem cells is able to stratify breast cancers (basal tumors from the other subtypes) and that CD49F^+^/DLL1^H^/DNER^H^ cells but not DNER^L^, both from poorly and well-differentiated tumors, are enriched in stem cells, as they can generate PD3D cell cultures and tumors in immunodeficient mice. Moreover, Tosoni et al. (2015) have recently shown that Numb is crucial for the maintenance of human mammary stem cell identity in the process of asymmetric cell division and progenitor maturation, and the loss of this gene induces EMT, resulting in the reacquisition of stemness characteristics. Recently, Tosoni et al. (2017), using breast cancer PD3D cell culture models and PDX, have demonstrated the efficiency of the inhibitor Nutlin-3, alone and in combination with chemotherapy, in inhibiting Numb^-^ tumor growth, metastasis, and tumor relapse *in vivo*, suggesting this inhibitor as an effective drug to selectively target CSC.

Huang et al. (2015) have generated 3D cultures from primary human pancreatic adenocarcinoma. These organoids recapitulate the histology architecture, express the same differentiation markers, and maintain the phenotypic heterogeneity of patient tumors. Moreover, they generate tumors when engrafted in immunodeficient mice, showing also a retention of the stem cell compartment. Recently, to study the role of the epigenome in the maintenance of the stem cell compartment of pancreatic ductal adenocarcinoma, Zagorac et al. (2016) have analyzed the DNA methylome in pancreatic CSCs. Using pancreatic cancer PD3D models, the authors demonstrated that the DNA methyltransferase is crucial to the maintenance of the self-renewal feature among CSCs, suggesting that the targeting of epigenetic regulators could improve therapy efficacy in pancreatic cancer patients.

## *In Vitro* 3D Models Simulating Tumor Microenvironment Heterogeneity for the Study of Tumor Progression and Sensitivity to Treatment

**FIGURE 2:**
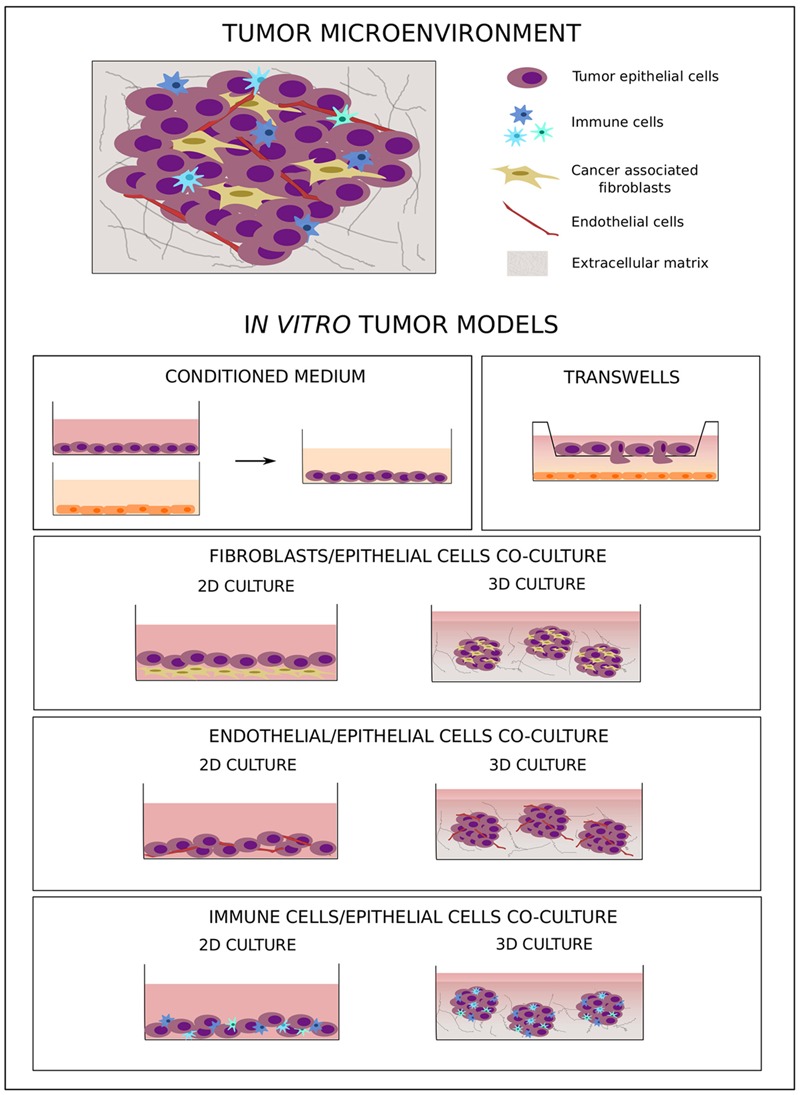
*In vitro* co-culture models of tumor–microenvironment interaction. The cartoon shows the main *in vitro* 2D and 3D co-culture models used to study tumor–microenvironment crosstalk.

Tumor development and metastatic progression are not isolated processes simply driven by survival and proliferation of altered epithelial cells; rather, they are complex mechanisms characterized by the dynamic interaction of neoplastic cells with components of the surrounding microenvironment, such as soluble factors, fibroblasts, tumor vasculature, and immune cells (**Figure 2**) (Mueller and Fusenig, 2004; Tlsty and Coussens, 2006; Hu and Polyak, 2008). Under healthy conditions, the microenvironment represents a barrier against tumor development (Dolberg and Bissell, 1984; Barsky and Karlin, 2005; Polyak and Hu, 2005). The presence of abnormal cells induces critical changes in the mechanical properties of the ECM (DuFort et al., 2011) as well as in the secretion of chemokines, cytokines, growth factors, and matrix remodeling factors (Allinen et al., 2004), which in turn activate a process of tumor–microenvironment co-evolution that sustains tumorigenesis (van den Hooff, 1988; Ronnov-Jessen et al., 1996).

Recently, Thery et al. (2002) have identified a mechanism of inter-cellular communication based on the secretion of extracellular vesicles involved in pre-metastatic niche formation. All cell lineages in the tissues as well as cancer cells can secrete into the extracellular compartment small vesicles (30–150 nm) called exosomes that carry a variety of molecules such as lipids, proteins, RNA, and DNA. Through this mechanism, cancer cells can modify the local microenvironment as well as systematically alter distant organ tissues, supporting the formation of the pre-metastatic niche (Azmi et al., 2013; Lobb et al., 2017). Since exosome content is strictly related to the cell of origin, exosomes represent an interesting tool for the identification of novel diagnostic and prognostic biomarkers (Xu et al., 2016).

The fundamental role of the cross-talk between tumor epithelial cells and the surrounding microenvironment was observed more than a century ago, when the seed and soil hypothesis was proposed (Paget, 1989). In particular, this model postulated that tumor preference for metastasis in specific secondary organs was due to both the intrinsic characteristics of specific cancer cells and a favorable interaction between tumor cells (the “seed”) and the organ microenvironment (the “soil”). Moreover, it has been demonstrated that tumor cells in the primary site can directly activate hematopoietic progenitors that migrate from the bone marrow to the future metastatic site, creating a suitable milieu for metastatic spreading (Kaplan et al., 2005, 2006).

A fundamental step for tumor progression is the dissemination of malignant cells to distant organs through blood circulation. It has been shown that CTCs are present in the blood stream of tumor patients already at early stages, when the presence of distant metastasis is still not clinically detectable (Braun et al., 2005; Sanger et al., 2011). Since CTCs can be easily collected with low invasive methods and represent specific tumor sub-clones with the ability to migrate and survive in the blood stream and to eventually reach and colonize secondary organs, their importance as prognostic and predictive biomarkers is clear (Mocellin et al., 2006; Rahbari et al., 2010; Miyamoto et al., 2012; Zhang et al., 2012), as is their promise in the study of both spatial and temporal tumor heterogeneity (Babayan et al., 2013; Heitzer et al., 2013; Mostert et al., 2013). Due to the high potential of CTCs not only for use in biomarker characterization but also as experimental models for the study of tumor progression and treatment susceptibility, several research groups have developed new methods for *in vitro* culturing of CTCs (Khoo et al., 2015; Vishnoi et al., 2015). Zhang et al. (2013) isolated CTCs from breast cancer patients and developed long-term *in vitro* cell lines based on a two-step cultivation method from 3 out of 38 patient samples. The molecular characterization of the cell lines highlighted a potential signature of brain metastasis based on the expression of the four markers HER2^+^/EGFR^+^/HPSE^+^/Notch1^+^. Moreover, the authors demonstrated the ability of CTCs expressing these markers to metastasize to the lung and brain after injection in immunodeficient mice.

In a proof-of-concept study, Yu et al. (2014) demonstrated the feasibility of using CTC-derived cell lines when selecting the best therapeutic options for individual cancer patients. In this study, the authors showed that non-adherent and hypoxic (4% CO2) culture conditions strongly improved *in vivo* proliferation abilities of CTCs isolated from estrogen receptor positive breast cancer samples. Interestingly, only CTCs isolated from recurrent patients formed long-term *in vitro* cell lines, suggesting a correlation between tumor susceptibility to treatment and CTC status. Finally, a different sensitivity to drugs targeting ER, PI3KCA, and HSP90 was shown, demonstrating the potential of using CTC-derived cell lines when defining new therapeutic targets for individual tumors. The ability of CTCs from patients with breast cancer to grow as 3D spheroids has been also demonstrated by Vishnoi et al. (2015), who isolated the CTC fraction positive for the stem cell signatures of CD44^+^/CD24^-^. The potential of using CTC-derived *in vitro* cell lines as experimental models has been proven not only in breast cancer but also in other tumor types, such as prostate cancer (Gao et al., 2014), colon cancer (Cayrefourcq et al., 2015; Grillet et al., 2017), and gastric cancer (Kolostova et al., 2016).

Considering the fundamental role tumor microenvironment plays in cancer development as well as in chemo-resistance, new therapeutic approaches have been developed to target not only tumor cells but also components of this supporting environment (Loeffler et al., 2006; Rossler et al., 2008; Dougan and Dranoff, 2009). Due to the heterogeneous composition of tumor tissues, more complex preclinical models are needed to better simulate human tumor biology. In this context, *in vitro* models, even as simplified representations of the complex cancer biology, are flexible tools that allow study of the crosstalk between different tumor components under controlled experimental conditions. To this aim, several co-culture models with different levels of complexity have been developed. The simple use of a conditioned medium (**Figure 2**) allows analysis of the effect of soluble factors on cell behavior, while migratory and invasive abilities can specifically be studied using transwell systems with different cell types plated in separate compartments (**Figure 2**) (Boyden, 1962). These two approaches are simple and easy to handle, but they do not allow study of cell–cell and cell–microenvironment interactions. To better understand the role of direct crosstalk between cells in cancer development and chemo-resistance, several co-culture systems have been developed where different cell types are simultaneously grown in direct contact with one another (**Figure 2**). To study the effect of tumor microenvironment on treatment resistance, Straussman et al. (2012) have analyzed the ability of stroma cells to affect the drug sensitivity of cancer cells, demonstrating that the efficacy of anti-cancer drugs in inducing cell death was reduced in the presence of stromal cells. Moreover, this effect was more pronounced when, in comparison to chemotherapy, target agents were used, highlighting the importance of the tumor microenvironment in influencing intracellular molecular pathways.

Even if direct co-culture models better represent the complex nature of tumor tissue, one of the main limitations is represented by the fact that these were initially based on 2-dimensional cultures, which do not mimic the three-dimensionality of a tumor *in vivo*. The recent development of more complex three-dimensional (3D) co-culture methods has allowed researchers to study tumor biology in a system that more closely resembles the physiological situation. Cancer-associated fibroblasts (CAFs) are altered fibroblasts actively involved in tumor development and progression, and a correlation between their abundance and poor prognosis has been demonstrated in several cancer types (Fujita et al., 2010; Yamashita et al., 2012). Considering their fundamental role in tumor progression (Bhowmick et al., 2004; Kalluri and Zeisberg, 2006), several tumor epithelium-fibroblast co-culture models have been developed. Li and Lu (2011) co-cultured 4T1 cells with murine embryonic fibroblasts (MEF) in a 3D system. To optimize spheroid formation, different protocols were compared, demonstrating the critical effect of the presence of a basement membrane in the form of Matrigel not only in the pre-coated wells but also in the culture medium. The incorporation of fibroblasts led to the formation of “polarized-like” alveolar and ductal structures similar to those observed *in vivo*, the morphology of which was influenced by the amount of fibroblasts present, with ductal networks developed only with a higher percentage of MEFs (above 50%). Other groups have likewise studied the effect of co-cultures on breast cancer progression (Jaganathan et al., 2014). Rama-Esendagli et al. (2014) used a three-component system comprising 4T1 breast cancer cells, NIH/3T3 and 3T3-L1 fibroblasts, and J774A.1 macrophages to show that breast cancer cell spreading and invasion were primarily supported by fibroblasts, while the presence of both fibroblasts and macrophages increased the capacity for 3D growth.

Another component of the surrounding microenvironment that strongly influences tumor cell survival and sensitivity to treatment as well as the ability to metastasize is the vascular network. Formation of new vessels and modification of the existing vascular network within a tumor are well-known processes allowing tumor cells to adapt to a constantly transforming environment (Chang et al., 2000; Hillen and Griffioen, 2007). The variability in this network, which supports the delivery not only of nutrients and oxygen but also of drugs to tumor cells, can therefore contribute to phenotypic heterogeneity, such as different tumor cell proliferation areas inside the tumor mass and differential responses to therapy (Tong et al., 2004; Multhoff and Vaupel, 2012; Fan et al., 2013). Chwalek et al. (2014) have successfully used *in situ*–forming starPEG-heparin hydrogels containing covalently bound integrin ligands and reversibly conjugated pro-angiogenic growth factors to develop a well-organized endothelial capillary network. The authors demonstrated the feasibility of applying this system for the study of heterotypic cell–cell interactions during tumor vascularization by transferring pre-formed hepatocarcinoma cell spheroids into hydrogels containing endothelial cells and analyzing the ability of these cells to migrate inside the tumor spheroids.

Recently, the effect of tumor microenvironment manipulation on cancer progression was analyzed using a complex 3D model where HT29 and HCT116 colorectal adenocarcinoma cells were co-cultured with human dermal fibroblasts (HDFs) and human umbilical vein endothelial cells (HUVECs). In this work, Magdeldin et al. (2017) demonstrated that ECM density strongly influenced cell migration patterns and that laminin, one of the components of the basement membrane, was involved in the regulation of endothelial cell morphology and vascular network formation. Moreover, they showed the simultaneous presence of glandular structures, polarized collective migration, and elongated cells during tumor cell migration, suggesting the involvement of several distinct mechanisms in this process. Interestingly, 3D tumor spheroids with a size bigger than 500 μm mimic themselves the typical layer-like structure observed in a tumor mass with an inner necrotic core due to the presence of hypoxic areas, surrounded by a middle quiescent part and an outer layer of proliferating cells (Mueller-Klieser, 1984; Carlsson and Acker, 1988; Alvarez-Perez et al., 2005).

The immune system plays a fundamental role in preventing tumor development and progression by recognizing and eliminating aberrant cells. However, under constant immune pressure, neoplastic cells can evade immune surveillance and instead co-opt immune cells to favor their sustained proliferation (Kim et al., 2007). Pioneering work by Sutherland et al. (1977) demonstrated that allo-specific spleen cells were able to infiltrate tumor spheroids and kill cancer cells. Since then, several research groups have focused on the development of new therapeutic approaches that take advantage of the cytotoxic effect of the immune system. In this context, several co-culture models of tumor cells with components of the immune system have been developed. By co-culturing HT29 cells with THP1 macrophages in the presence of immunostimulants such as heteroglycans or LPS, Devi et al. (2015) have demonstrated the efficacy of activated macrophages in killing tumor cells. The cytotoxicity not only of macrophages but also of other immune cells such as NK cells (Giannattasio et al., 2015) monocytes (Konur et al., 1996, 1998), and leukocytes (Hirschhaeuser et al., 2009) has also been studied.

In the study of tumor progression as well as response to treatment, selection of the most appropriate model is essential since the composition of the *in vitro* system can strongly influence the result of the analysis. Recently, Lal-Nag et al. (2017) compared the effects of different experimental models on ovarian cancer drug sensitivity. In particular, they developed a 3D model comprising ovarian cancer cells alone or co-cultured with the omentum metastatic niche, and they demonstrated that different microenvironments strongly influence response to treatment. Since development of distant metastasis is the main cause of death among cancer patients, experimental models that mimic the metastatic process are strongly needed to allow for the study of crosstalk between tumor cells and the tumor microenvironment. Recently, Holen et al. (2015) developed a humanized bone model to study breast cancer metastasis. In particular, they used trabecular bone cores obtained from the femoral heads of patients undergoing hip replacement surgery to seed the human breast cancer cell lines MDA-MB-231 and T47D. The use of bone disks allows for the maintenance of all cell types present in the bone and involved in breast cancer colonization, making these disks a perfect model for the study of breast cancer bone colonization and metastatic development. A similar approach has been used to culture breast and prostate cancer cells, isolating bone tissues from female and male patients, respectively (Salamanna et al., 2016).

Among bone marrow components, adipocytes play a critical role in bone metastatic progression. To study the interaction between tumor cells and adipocytes, Herroon et al. (2016) have developed a transwell-based *in vitro* model co-culturing prostate tumor cell spheroids in the presence of bone marrow–derived adipocytes. The constant exposure to soluble factors produced by adipocytes allows for the analysis of their influence on prostate cancer cell behavior. Moreover, the authors directly co-cultured the two cell types to study the mechanism of tumor cell invasion toward the adipocytes. Adipocytes and tumor cells were separated by a layer of collagen I, and a time course analysis allowed for definition of the ability of cells to invade the collagen matrix and for examination of how this is influenced by adipocytes.

Hoffmann et al. (2015) have studied the sensitivity of different colorectal cancer models to clinically relevant combination therapies and to targeted therapeutics. In particular, they compared the drug response of colorectal cancer cell line grown as spheroids comprising tumor cells alone or tumor cells co-cultured with peripheral blood mononuclear cells (PBMCs) or with primary cancer-associated fibroblasts to the response of spheroids directly isolated from colon cancer tissues. After treatment, an alteration in cell response was observed when diverse tumor microenvironments were used. Moreover, distinct response patterns not detectable in the cell line–based models were revealed in the patient-derived spheroids. Taken together, these data demonstrate the complexity of the influence of tumor microenvironment on drug sensitivity. Even considering the difficulty of obtaining fresh tumor tissue and the intrinsic patient to patient variability in drug response, cancer spheroids directly isolated from fresh tumor tissues with preservation of cellular heterogeneity represent the most promising preclinical model for drug screening.

## Patient-Derived Xenografts Recapitulating Intra-Tumor Heterogeneity

It has been demonstrated that PDXs preserve the morphology and 3D architecture found in original human tumor tissue. Nevertheless, the interaction of human cells with the murine microenvironment could alter cell features and behavior as a result of interspecies (in)compatibility. In the history of preclinical models, patient-derived xenografts pose a significant advantage over classical cell line models (Hidalgo et al., 2014; Aparicio et al., 2015; Byrne et al., 2017). PDX models autonomously recapitulate much of the inter- and intra-tumor heterogeneity of the donor tumor tissue (Nguyen et al., 2014; Eirew et al., 2015), which make them the gold standard for oncologic drug discovery and preclinical development. At the same time, they can be used to study resistance to chemotherapy through the use of isogenic cellular clones, as shown in colorectal PDX models (Kreso et al., 2013).

As explained above, clonal populations within the tumor exist dynamically in time and space. Three main approaches are used to describe clonal dynamics. Phenotypic clones are investigated by lentiviral barcoding, mutational clustering by following genomic clones, and single cell–based by computational approaches (Fisher et al., 2013; Eirew et al., 2015). For example, Ding et al. (2010) have found that PDX models established from a basal-like breast cancer are more representative of a patient’s metastatic lesion than of the primary tumor. Using a Bayesian clustering method for grouping somatic mutations, Eirew et al. (2015) have likewise reconstructed the genomic clonal dynamics of breast cancer PDX models and found that in each of the 15 matched tissues examined, clonal diversity was reduced in the PDX. Similar findings have been presented by Schutte et al. (2017) for colorectal tumors and derived models, where the latter reflected only a subset of consensus groups. Reasons for these findings may be manifold, ranging from engraftment bias to minor clones restricted to only small areas in the sample of origin. Remarkably, similar clonal dynamics were observed in matched xenografts and PD3D cultures established from the same sample (Schutte et al., 2017). These examples suggest that micro-environmental, stromal, and immune compartments on one hand and deterministic mechanisms on the other underlie the clonal selection found on engraftment. In conclusion, PDX models partially recapitulate the complexity of clonal dynamics observed in human cancers. Biases caused by the engraftment can act as non-stochastic selection events, which can specifically define a patient-derived model more than limit it.

Clonal evolution is a complex process. During therapeutic intervention, selective pressure may substantially alter a heterogeneous composition. This may result in the need for multiple-site repeated biopsies to decipher the clonal composition in different regions of the tumor for evidence-driven support of clinical decisions. This procedure would be highly invasive, technically not applicable and prevented by the current standard pathology routine. With this in mind, PD3D models in combination with liquid biopsies (Aparicio and Caldas, 2013; Murtaza et al., 2013) may prove useful. In addition to manifested genomic aberrations, aberrant DNA methylation patterns in cancer can blur the lines between distinct phenotypic “attractor states” or “cancer stem cells” (Marusyk et al., 2012), making them drivers of malignant progression independent of their genetic background. To track isogenic cellular clones in various cancer PDX models, including colorectal cancer, lentiviral tagging has been used to barcode individual cells and their progeny. It has been found that relatively rare quiescent sub-clones (putative cancer stem cells) in colorectal PDXs are able to recapitulate the entire tumor bulk heterogeneity after treatment with chemotherapy (Kreso et al., 2013; Nguyen et al., 2014). Moreover, it has been shown that the genetic background of these quiescent cell populations is similar to their highly proliferative counterparts. In a follow-up publication, they were linked to the stem cell BMI1^+^ population present in the intestinal and colonic crypts (Kreso et al., 2014). By definition, these cellular clones were isogenic, so it can be speculated that microenvironmental factors may have regulated cellular plasticity by supporting distinct gene expression patterns or epigenetic attractor states (Michor and Weaver, 2014). Hence, the microenvironment’s composition in a PDX could profoundly alter the phenotype of donor cells, rendering drug sensitivity experiments difficult to interpret.

Matrigel is often used as a scaffold to increase engraftment efficiency in PDX models; however, for more representative results, this murine basement membrane extract is usually replaced by suitable synthetic alternatives, which have a defined presence of growth factors, structure, and protein composition, mimicking tissue-specific ECM, where needed (Cassidy, 2014). It has been shown that following tumor tissue xenotransplantation, murine stromal cells progressively replace corresponding human cells. Despite some differences between the ligand repertoires of donor and host fibroblasts, both types of fibroblasts are able to interact with cancer cells (Argent et al., 2010). How this partial compatibility reflects the patient’s stroma with regard to stroma composition and its influence on tumor growth and development remains elusive. To reduce the influence of murine stroma on the PDX model, many have begun to explore co-engraftment of human mesenchymal stem cells (MSC) or CAF cell lines in PDXs. Resistance to cytotoxic therapies among these cell lines is variable, and it is controlled by the intracellular hepatocyte growth factor (HGF)/c-Met signaling (Argent et al., 2010; Straussman et al., 2012). One promising approach is the direct isolation of patient-derived fibroblasts and the co-engraftment of these matched stromal components. This would significantly increase the advantages these models already have in retaining the complex heterogeneity found in patient samples.

The most obvious bias of using PDX models to study human tumor progression is the lack of an immune component in mice. The consequence of genetic alterations accumulated during tumor evolution is also strictly linked to the propagation of a large repertoire of neoantigens. The main focus of immunotherapy is to target these neoantigens. In the most straightforward approach, significant CD8^+^ cytotoxic T-cell infiltration would lead to tumor cell death. However, during progression, most tumors eventually evade the immune system. For that reason, co-engraftment of immune components into PDX models could help basic research to better understand patient-specific crosstalk of tumor progression and immune surveillance at different stages of the disease and furthermore help in the designing of novel therapies targeting tumor–immune interactions. The co-engraftment of human bone marrow stem cells with liver and thymus tissues provides a solid method to reconstitute the human immune system in immunodeficient mice (Shultz et al., 2007). However, the complexity needed to generate this modelmakes it largely unfeasible in clinical settings. On the other hand, the use of transplanted CD34^+^ hematopoietic stem cells in immunocompromised mice seems to be a suitable and feasible system to generate patient-matched humanized PDX (huPDX).

## Conclusion and Perspective

As our knowledge on cancer development and disease progression grows, the demand for reliable models increases. Simple modeling of pathway activation using monoclonal adherent cell lines is no longer sufficient when it comes to predicting therapeutic responses in 3D tissue. Within the tumor, cooperation and competition between genetically and phenotypically distinct sub-clonal populations seem to drive tumor growth, resistance to therapy, and recurrence. Today, an entire toolbox of preclinical *in vitro, in vivo*, and *in silico* models is available to unscramble the complexity of ITH. All of these models have utility in basic cancer research and to a certain degree have demonstrated clinical predictive power. PD3D models can be reliably generated directly from the tumor tissue of either a primary or a metastatic lesion. The high degree of flexibility of PD3D cell cultures resides in their self-organizing nature; in their easy molecular manipulation, including CRISPR-mediated gene editing; and in their suitability for high-throughput drug screening. Moreover, PD3D cell culture models are able to maintain both the functional cell hierarchy and the polyclonal architecture of tumor biopsies, and when derived by multi-regional sampling at different time points, they can represent a reliable model to study tumor evolution in time and space, resulting in a deeper understanding of how to exploit these mechanisms for therapeutic intervention in clinical scenarios. Nevertheless, the development of more complex PD3D cell culture models, including cellular components of the microenvironment to mimic *in vitro* the interplay of immune and other stromal cells with the tumor in response to therapies, would be necessary to increase the predictive value of such models. Moreover, in our opinion, major efforts are needed to correlate the abilities of PD3D cell cultures with reliable drug responses observed in cancer patients, with the aim of integrating such data into the clinical decision-making process where possible.

Advancements in xenograft models, such as co-culture models of human tumors with human stroma and/or immune cells, make PDX models an ideal extension for *in vitro* experiments. These co-models recapitulate the complexity of human malignancy remarkably well with regard to the interaction with stromal or immune components. Nevertheless, as our understanding of graft vs. host interactions remains limited, data from these models must be interpreted with care. With the advent of systems biology, all existing data on clonal evolution in patients and matched *in vitro* and *in vivo* will be used to train algorithms to better model treatment response. In consequence, the coalescence of these approaches will bring a better understanding of the evolution of tumors and of their interaction with the (micro)environment. In the future, more proactive models of intra-tumor heterogeneity for drug development and treatment strategies will emerge.

## Author Contributions

Conceptualization, investigation, and writing: AS, CR, GG, MG, and UK. Funding acquisition: CR and UK.

## Conflict of Interest Statement

CR and AS are employees of cpo GmbH, a company involved in developing preclinical models. The other authors declare that the research was conducted in the absence of any commercial or financial relationships that could be construed as a potential conflict of interest.
